# Large granular lymphocytic leukemia cured by allogeneic stem cell transplant: a case report

**DOI:** 10.1186/s13256-022-03447-y

**Published:** 2022-06-08

**Authors:** Edward Carey, Nicholas Ward, Maher Abdul-Hay

**Affiliations:** 1 Department of Internal Medicine, New York University Grossman School of Medicine, 240 East 38th street, 19th Floor, New York, NY 10016 USA; 2 Department of Pathology, New York University Grossman School of Medicine, New York, NY USA; 3 New York University Perlmutter Cancer Center, New York, NY USA

**Keywords:** LGL, Allogeneic stem cell transplant, Neutropenia, GVHD

## Abstract

**Background:**

Large granular lymphocytic leukemia is a rare lymphocytic neoplasm that can pose a treatment challenge in patients with severe neutropenia in whom conventional therapies fail. We report one of the first cases in which allogeneic stem cell therapy was used as treatment for large granular lymphocytic leukemia.

We report and discuss the case of a 42-year-old white Caucasian female who, despite multiple therapies including methotrexate, cyclophosphamide, prednisone, cyclosporine, and pentostatin, continued to show severe neutropenia and recurrent infections. The patient was treated successfully and cured by allogeneic stem cell transplant without any major complications.

**Conclusions:**

The significant importance of this case report is the introduction of a new treatment algorithm for challenging cases of T-cell large granular lymphocytic leukemia in which standard care fails. We hope that this case report will raise awareness of the potential benefits of allogeneic stem cell transplant in the treatment of aggressive forms of T-cell large granular lymphocytic leukemia.

## Introduction

Large granular lymphocytic (LGL) leukemia is a rare lymphocytic neoplasm that can be divided into T-cell and natural killer cell LGL. T-cell LGL (T-LGL) is the most common form of the disease, representing about 85% of cases, and is the focus of our paper [1].

LGL is characterized by clonal production of T-cells and subsequent infiltration of the liver, bone marrow, and/or spleen [2]. It can cause cytopenias and have autoimmune manifestation. Median patient age at diagnosis of LGL is 66 years of age, with about 14% of patients being diagnosed before age of 50 years [2]. LGL leukemia is generally an indolent condition, but can be aggressive in some cases [3]. Treatment often involves supportive care, cyclophosphamide, methotrexate, and/or cyclosporine [4]. There is no clear consensus or guidance for care beyond first-line treatment.

Here, we describe the unique case of a relatively young woman with T-cell LGL (T-LGL) leukemia resistant to prior treatments. She had persistent and severe neutropenia, with absolute neutrophil count (ANC) of 0. Because of these factors, she underwent allogeneic bone marrow transplant. This is a unique case because it describes a rare condition, T-LGL leukemia, being treated with a rare modality, allogeneic bone marrow transplant. There have been few case reports about the use of stem cell transplant in LGL leukemia [5, 6].

However, what makes this case particularly unique is her ANC of 0 and subsequent graft versus host disease (GVHD) prevention after transplant. This is one of the first such cases reported in the USA, and we hope that this case report will raise awareness of allogeneic bone marrow transplant as a possible treatment for refractory T-LGL leukemia.

## Case

Our patient was a healthy white Caucasian woman until age 33 years, around 2009, when she developed oral ulcers and was tentatively diagnosed with lupus at another facility. She had no rash, and her antibody status is unclear. She was started on methotrexate, prednisone, and etanercept, but dosing and regimen are somewhat unclear. There are also notes that she received belimumab for lupus. Unfortunately, we do not have further data on how this diagnosis was reached or how firm it was. The first examination in our system in 2014 noted normal blood pressure and normal temperature, with normal heart rate and respiratory rate. She was ECOG 0, and had normal cardiopulmonary, abdominal, and skin examinations.

Before this, she had no major medical history. She had spine surgery over the course of her illness due to prolonged corticosteroid usage. She was pregnant once and had one healthy daughter. She was born in Russia, and did not report any toxic or occupational exposure. She worked as a speech therapist. She occasionally used alcohol and was a former smoker, with an approximately 5-year smoking history. She had no notable family medical history.

**Fig. 1:**
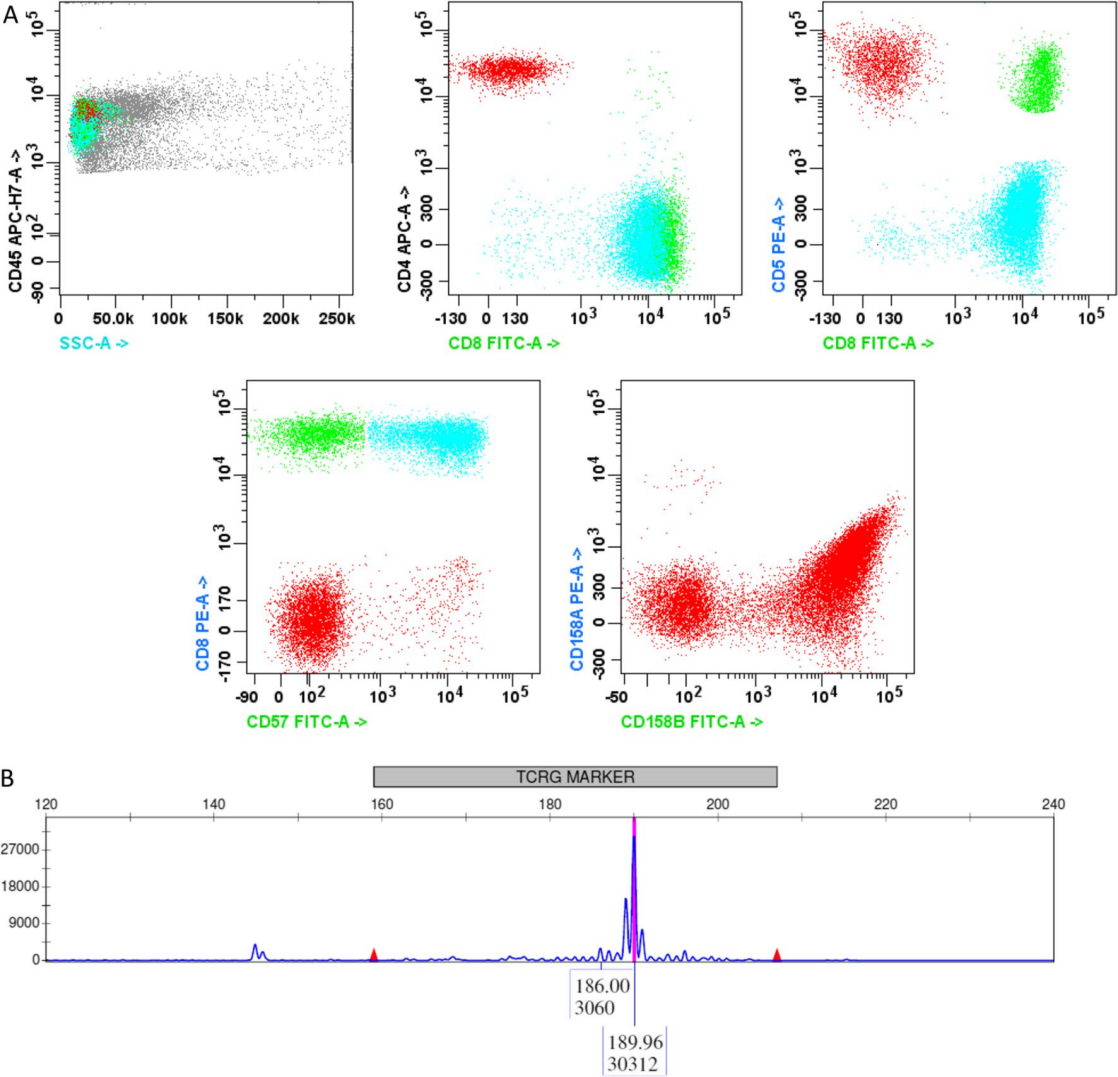
Flow cytometry showing a population of atypical T-cells (48% of total cells, 76% of total T-cells), highlighted in cyan expressing CD3 and CD8, but lacking CD4, CD5, and CD57. Analysis of KIR expression reveals the cells to preferentially express CD158b isoform; CD158a and CD158e (not shown) were not expressed. Normal background CD4^+^ and CD8^+^ T-cells are colored in red and green, respectively (**A**). Monoclonal T-cell receptor gamma gene rearrangement detected by PCR and capillary electrophoresis showing a single prominent amplicon present in a minimal polyclonal background. *X* axis = amplicon size. *Y* axis = fluorescence (**B**)

**Fig. 2:**
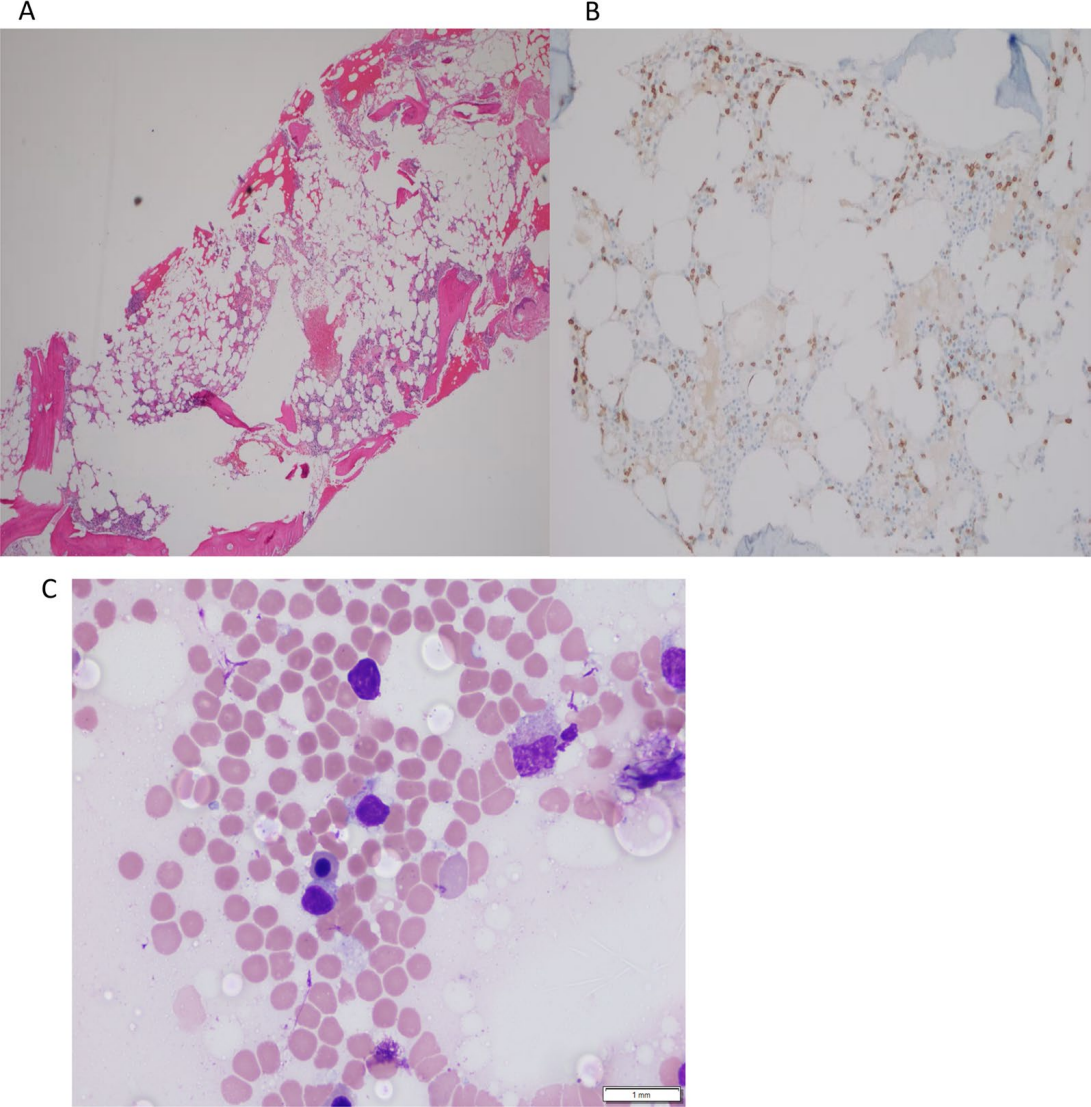
Bone marrow biopsy core showing hypocellular marrow (**A**), CD3-positive cells in marrow **B** and peripheral blood smear (Wright’s stain, 100×) featuring occasional intermediate-sized lymphocytes with round/slightly indented nuclei with inconspicuous nucleoli, condensed chromatin, and abundant pale cytoplasm with azurophilic granules (**C**)

She had persistent iron deficiency and neutropenia. In fall of 2014, she was diagnosed with LGL leukemia via flow cytometry. The first laboratory workup in our system was from fall of 2014, with a white blood cell count (WBC) of 3.3, with 17% granulocytes, 73% lymphocytes, hemoglobin 10.8, with mean corpuscular volume (MCV 76), and platelets of 209. In fall of 2014, she had negative tissue transglutaminase immunoglobulin A (IgA) antibody, negative rheumatoid factor, negative anti-nuclear antibody, and negative hepatitis C testing. Peripheral blood flow cytometry in December 2014 showed neutropenia associated with 22% atypical CD8^+^ T lymphocytes exhibiting an immunophenotype of CD3^+^ and CD57^+^ with partial loss of CD5 and CD7, consistent with T-cell LGL leukemia(Fig. 1A). Testing was positive for T-cell receptor gamma chain monoclonal gene rearrangement ( Fig. 1B). Her bone marrow core biopsy showed a hypocellular marrow ( Fig. 2A), and peripheral smear showed population of atypical T-cells, consistent with large granular lymphocytes (Fig. 2C).

She had recurrent infections, and in 2015, was found to have ANC of 0. She was started on oral methotrexate and steroid taper in early 2015, at 15 mg per week. After 6 months, due to persistent neutropenia, she was switched to oral cyclophosphamide 100 mg daily, with 20 mg prednisone. In fall 2015, cyclophosphamide was stopped and prednisone was increased. Subsequently, in early 2016, she received two cycles of pentostatin, 6.2 mg, IV and 100 mg of cyclosporine twice per day. Neither of these therapies improved her neutropenia. Despite the failure of these therapies, her neutropenia responded and improved to prednisone, but would recur and worsen when tapered. She had pathologic fractures requiring surgery due to prolonged steroid exposure. She continued on prednisone for 2 years but had persistent severe neutropenia and osteoporosis. She was evaluated but did not qualify for a clinical trial due to her ANC of zero. Further investigations in 2016 showed anti negative double-stranded DNA antibodies, negative anti-Smith antibodies, negative glucose-6-phosphate dehydrogenase (G6PD), and negative cyclic citrullinated peptide (CCP) immunoglobulin G (IgG) antibody.

Peripheral blood flow cytometry was sent again in 2016, showing a similar aberrant CD8^+^ T-cell population to previous studies consistent with persistent T-cell LGL leukemia. Extracted genomic DNA was analyzed for T-cell receptor gamma chain gene rearrangements and again was positive for monoclonal T-cell receptor gamma chain rearrangement. DeZern and Sekeres [7] argue in their paper that peripheral flow cytometry is the most accurate way to detect LGL leukemia, with cells being CD3^+^, CD5 dimly positive, and CD57 positive, and argue for determining clonality in T-cell gene rearrangements. Our patient’s sample showed 38% of T-cells as CD8^+^, CD3^+^, and CD57^+^, consistent with T-LGL leukemia.

Bone marrow biopsy done at an outside facility described a nearly identical aberrant CD8^+^ T-cell population consistent with T-cell LGL leukemia with no features concerning for myelodysplasia or other malignancy.

Starting around age 42 years, around 2018, after additional infections including pneumonia, persistent severe neutropenia, and treatment failure despite multiple different therapies, she was referred for bone marrow transplant evaluation in fall of 2018. Her brother was a full human leukocyte antigen (HLA)-matched sibling and was her donor. She had abdominal imaging, showing no acute abdominal pathology, without splenomegaly or lymphadenopathy noted. A repeat bone marrow biopsy showed persistent T- LGL leukemia (48% involvement by flow cytometry), with background 10% cellularity featuring marked granulocytic hypoplasia with no features concerning for myelodysplasia. Genomic DNA was analyzed for T-cell receptor gamma gene rearrangements, and was again positive. Cytogenetics showed chromosome 17 monosomy in 5% and p53 deletion in 10% of nuclei seen, and they were unlikely contributing for her underlying LGL leukemia.

At age 42 years, in 2019, she was admitted for bone marrow transplant. On admission she was afebrile with normal heart rate and blood pressure. Physical examination was notable in that she was alert and oriented, with normal cardiopulmonary examination. She had no cranial nerve deficits. She had no rashes. She underwent a reduced intensity conditioning regimen with fludarabine 30 mg/m^2^, days −7 to −2, busulfan 130 mg/m^2^ on days −3 and −2, and rabbit anti-thymocyte globulin on days −4 to −2. She successfully received allogeneic bone marrow transplant from her brother. After her transplant, she received mycophenolate 1000 mg every 8 hours and tacrolimus twice per day for GVHD prophylaxis.

Shortly after transplant, while still hospitalized, she had a brief neutropenic fever, which was treated with antibiotics. She had a negative infectious workup and remained afebrile and stable. She was discharged on tacrolimus, mycophenolate, acyclovir, and trimethoprim/sulfamethoxazole. Overall, she tolerated the transplant well with no major complications nor GVHD.

She continued mycophenolate until day 28, and continued tacrolimus until 5 months posttransplant. Bone marrow biopsy months after transplant, in July 2019, showed full chimerism, with a normal male karyotype and hypocellular trilineage hematopoiesis with no flow cytometric evidence for persistent T-cell LGL leukemia. T-cell receptor monoclonal rearrangements were reported negative. Laboratory workup at about 20 months posttransplant showed normal white blood cell count, with recovery of her ANC to normal, and no anemia. Platelet count was normal. As of submission of this manuscript, she continues to enjoy normal ANC and no GVHD at about 2 years from her stem cell transplant.

The patient was pleased with the outcome of her stem cell transplant and the recovery of her ANC. She felt overall well and tolerated the procedure without any significant adverse events or complications. As noted above and later in our discussion [5, 6, 8], allogeneic bone marrow transplant can be an effective treatment for severe cases of LGL leukemia.

## Discussion

LGL leukemia can be divided into T and NK cell associated diseases, with T-cell disease being significantly more common and less aggressive than NK cell disease [1, 9–11]. T-LGL leukemia, as noted above, is a rare lymphoproliferative disorder with variable but generally indolent course [2, 3, 12]. In this condition, CD3^+^/CD8^+^ T lymphocytes proliferate and can infiltrate bone marrow, liver, and spleen [2]. Symptoms are often due to anemia and neutropenia, as well as autoimmune features and arthralgias [4]. This may explain our patient’s preliminary diagnosis of lupus. These patients can also have persistent lymphocytosis, hypergammaglobulinemia, and splenomegaly [9]. The majority of patients with T-LGL leukemia will eventually need some treatment, which is often immunosuppressive [4].

As noted above, what makes this case particularly unique and notable is the aggressiveness of her disease and profound and persistent neutropenia to 0. Furthermore, the GVHD prevention after transplant was a novel approach. This is one of the first such cases of allogeneic stem cell transplant for T-LGL leukemia in the USA. This case is particularly remarkable given the severity of her neutropenia and multiple failed prior lines of treatment, and the effectiveness of stem cell transplant in treating this previously refractory disease. We hope to raise awareness of allogeneic bone marrow transplant as treatment for refractory T-LGL leukemia.

### Pathophysiology

Genome-wide mutational analysis of LGL leukemia cells appeared to be enriched in cellular signaling cascades that have been implicated in the abnormal survivability of the cells [3]. In particular, signal transducer and activator of transcription factor 3 (*STAT3*) is a mutation that has been described in approximately one-third to half of LGL leukemia cases [3, 13, 14]. Mutations in STAT5b have been associated with a more aggressive clinical course (PMID: 23596048). Blockage of interleukin 6 (IL6) has also been shown to return adequate LGL cell apoptosis, suggesting its involvement in LGL leukemia [3]. Additional pathways have been implicated, including MAPK, RAS-RAF1, MEK/ERK, and NFKB [3]. Shah *et al.* [9] also showed that LGL leukemia patients have dysregulation of Fas-mediated apoptosis. Gazitt and Loughran [15] described how neutropenia, as seen in our patient, is a fairly common sign of LGL leukemia. They also discuss how associated immunocompromise contributes to the morbidity and mortality associated with LGL leukemia. They discuss how LGL leukemia causes neutropenia through a few mechanisms, including hypoplasia, inhibitory cytokines, and elevated Fas ligand, which can cause apoptosis in neutrophils [16].

T-LGL is often a fairly indolent disease, but our case shows how it can be aggressive, progressive, and debilitating. Alekshun *et al.* [12] performed a review of 13 cases of aggressive T-cell LGL leukemias. They noted that most patients with aggressive disease were CD3^+^, CD8^+^, CD56^+^, and CD57^–^. However, our patient had CD57 positivity. They noted that patients with aggressive T-LGL leukemia had B-symptoms, lymphadenopathy, cytopenias, and splenomegaly.

### Standard of care therapies

Treatment for LGL leukemia often begins when patients develop severe neutropenia, symptomatic neutropenia, or constitutional symptoms due to transfusion-dependent anemia [1]. However, many patients do not require therapy [17]. Immunosuppression is the backbone of first-line LGL leukemia treatment, consistent with our understanding above that LGL leukemia represents constitutively activated lymphocytes [1]. This first-line treatment often involves methotrexate, cyclophosphamide, and cyclosporine [1]. Some reports note that cyclosporine or oral weekly methotrexate leads to responses in about 75% of cases [17]. Our patient underwent a fairly similar regimen, but unfortunately did not have a good response. Growth factors such as erythropoietin or granulocyte colony-stimulating factor (GCSF) can help to augment response to therapy. Patients need to undergo at least 4 months of treatment before assessing for response, via clinical and blood count assessment [1, 18]. It has been shown that about 50% of patients respond to these regimens, and all of these treatment options lead to fairly similar responses [1]. Second-line treatment is variable and can include purine analogs, such as pentostatin, which our patient received, and alemtuzumab [17, 3].

### Novel therapies

Some of the current research on LGL leukemia treatment has focused on novel therapies that are more targeted towards the disease’s pathogenesis. As noted above, *STAT3* has been implicated in LGL leukemia. The JAK inhibitor, tofacitinib, which works on the JAK-STAT pathway, was studied in a small population of nine patients with refractory T-LGL leukemia and associated rheumatoid arthritis [19]. In that trial, six of nine patients had hematologic response, and five out of seven had improvement in neutropenia. Moignet and Lamy noted promising results in trials of JAK/ STAT inhibitors and cytokine inhibitors for LGL leukemia.

There are also some data regarding alemtuzumab, a monoclonal antibody against CD52 that is often used in organ transplantation, as a potential treatment for LGL leukemia. Dumitriu and colleagues performed a phase 2 open-label study of alemtuzumab in patients with T-cell LGL leukemia [20]. This was a small but somewhat promising study, as 14 of 19 patients had positive response. However, alemtuzumab causes prolonged depletion of B and T lymphocytes, which can be both helpful and harmful [21]. The benefits of treatment with alemtuzumab may not outweigh the risks of profound and prolonged immunosuppression in more mild cases of T-LGL leukemia [21]. Alemtuzumab treatment is associated with viral reactivation, particularly of cytomegalovirus (CMV) [22]. This can have serious consequences for immunocompromised patients, as well as those anticipating or undergoing stem cell transplant.

### Stem cell transplant

Alekshun *et al.* [12] published a case report of a patient with aggressive T-cell LGL leukemia who underwent aggressive multimodal treatment and eventually autologous bone marrow transplant. Their patient presented with acute-onset B symptoms, anemia, low platelets, and hepatosplenomegaly. The malignant cells in his blood showed CD3^+^, CD8^+^, and CD56^+^, and T-cell receptors showed clonal rearrangement. He was diagnosed with aggressive T-LGL leukemia. He underwent treatment with cyclophosphamide, vincristine, doxorubicin, and dexamethasone (hyper-CVAD) alternated with methotrexate and cytarabine. He also received intrathecal methotrexate and cytarabine. GCSF and alemtuzumab were given to help mobilize peripheral blood hematopoietic cells. He then received carmustine, etoposide, cytarabine, and melphalan (BEAM) as a conditioning regimen before autologous stem cell transplant. This study showed that autologous stem cell transplant could be a treatment option for T-cell LGL leukemia. However, their patient’s neutropenia was not as severe as in the patient described heein. The British Society of Hematology’s 2011 guidelines for T-cell neoplasms note that patients with aggressive T- LGL leukemia should undergo more intensive combination chemotherapy, but they noted that there are insufficient data to support one regimen over another [17].

La Nasa *et al.* [6] published another case that demonstrated the role of stem cell transplant in LGL leukemia. They treated a 57-year-old man with LGL leukemia and multiple sclerosis (MS) with allogeneic stem cell transplant from a sibling who was an HLA-identical match. Their patient underwent a conditioning regimen with fludarabine, busulfan, and cyclophosphamide. He underwent GVHD prophylaxis with cyclosporine and methotrexate. He was doing well at 3 years of follow-up and had complete remission of his LGL leukemia. To note, his MS also improved after hematopoietic stem cell transplantation (HSCT). This also highlights the autoimmune manifestations of LGL leukemia.

In 2016, the European Society for Blood and Marrow Transplantation [8] published a review of stem cell transplant for T-LGL leukemia. They discussed escalating therapy with intensive chemotherapy and possibly stem cell transplant for aggressive cases. They described 15 patients who had either autologous HSCT or allogeneic HSCT for T-cell LGL leukemia. Five patients underwent autologous stem cell transplant, and ten underwent allogeneic stem cell transplant. A variety of conditioning regimens were used, including BEAM, cyclophosphamide with total body irradiation (CY-TBI), fludarabine, melphalan, and alemtuzumab, and others. There were also a variety of previous treatments for each of the patients. These data all indicate that there is no standardized way to treat LGL leukemia, especially more complicated, refractory, or aggressive cases. This also highlights the variety of ways to prepare patients for bone marrow transplant for this condition. Thus, all of this only indicates the need for further research on and awareness of bone marrow transplant, both autologous and allogeneic, to treat LGL leukemia.

Three of the patients in the above-mentioned review who had autologous transplant had complete response, and two had progression of disease. Three were alive at last follow-up. Of the ten patients who had allogeneic stem cell transplant, five had complete response, two had partial response, one had progression, one had relapse, and one did not have data on his disease response. Seven of the patients who underwent allogeneic stem cell transplant had GVHD. Five patients had acute GVHD, and two patients had chronic GVHD. Of the patients who had acute GVHD, two patients had grade 1, one had grade 2, one had grade 3 acute, and one had grade 4 disease. Two additional patients had what was described as extensive chronic GVHD. Five of the ten patients who underwent allogeneic stem cell transplant were alive at last follow-up. One patient died of fungal infection, another died of viral and fungal infection, one died of GVHD and viral infection, and another died of relapse. This shows that bone marrow transplant for LGL leukemia is not without risks.

Donato and colleagues also described their experience with two patients with T-cell LGL leukemia who they treated with reduced-intensity allogeneic stem cell transplant, similar to our case [5]. One of their patients was a 54-year-old woman with profound anemia and splenomegaly due to T-cell LGL leukemia. She underwent treatment with methotrexate, cyclosporine, equine antithymocyte globulin, and alemtuzumab. She then underwent a fludarabine and melphalan conditioning regimen before a related donor stem cell transplant. The other patient was a 34-year-old woman with T-cell LGL leukemia and associated lymphocytosis and infections. She failed treatment with a splenectomy and cyclosporine. She underwent conditioning with rabbit anti-thymocyte globulin, fludarabine, and melphalan, before nonrelated donor stem cell transplant. Both patients underwent GVHD prophylaxis with tacrolimus and methotrexate. The latter had stage 1 skin GVHD. Both patients achieved complete remission, and at the time of that publication both were in complete response greater than 1 year after transplant.

## Conclusion

This case demonstrates some of the presenting signs and symptoms of severe T-LGL leukemia. It highlights a refractory case made more difficult by neutrophil count of zero that was overcome by allogeneic stem cell transplant. We hope that this case report will help to highlight the pathogenesis and some of the emerging therapies to help treat this rare disease. What is notable about this case is how allogeneic stem cell transplant is underperformed for T-LGL leukemia. We hope to highlight how it can be indicated in certain cases such as ours with severe neutropenia. We hope that further research investigates allogeneic stem cell transplant to treat refractory cases of T-LGL leukemia, with a focus on an optimal conditioning regimen and GVHD treatment. We hope that future research will provide understanding of where allogeneic stem cell transplant should fit into the LGL leukemia treatment landscape.

## Data Availability

Supporting data are available and can be provided if required.
